# *In vivo* importance of heparan sulfate-binding glycoproteins for murid herpesvirus-4 infection

**DOI:** 10.1099/vir.0.005785-0

**Published:** 2009-03

**Authors:** Laurent Gillet, Janet S. May, Philip G. Stevenson

**Affiliations:** Division of Virology, Department of Pathology, University of Cambridge, UK

## Abstract

Many herpesviruses bind to heparan sulfate (HS). Murid herpesvirus-4 (MuHV-4) does so via its envelope glycoproteins gp70 and gH/gL. MuHV-4 gp150 further regulates an HS-independent interaction to make that HS-dependent too. Cell binding by MuHV-4 virions is consequently strongly HS-dependent. Gp70 and gH/gL show some *in vitro* redundancy: an antibody-mediated blockade of HS binding by one is well tolerated, whereas a blockade of both severely impairs infection. In order to understand the importance of HS binding for MuHV-4 *in vivo*, we generated mutants lacking both gL and gp70. As expected, gL^−^gp70^−^ MuHV-4 showed very poor cell binding. It infected mice at high dose but not at low dose, indicating defective host entry. But once entry occurred, host colonization, which for MuHV-4 is relatively independent of the infection dose, was remarkably normal. The gL^−^gp70^−^ entry deficit was much greater than that of gL^−^ or gp70^−^ single knockouts. And gp150 disruption, which allows HS-independent cell binding, largely rescued the gL^−^gp70^−^ cell binding and host entry deficits. Thus, it appeared that MuHV-4 HS binding is important *in vivo*, principally for efficient host entry.

## INTRODUCTION

Viruses must bind to cells to infect them. The binding specificities of herpesvirus glycoproteins should therefore be major determinants of tropism. Many herpesviruses bind to heparan sulfate (HS) (Shukla & Spear, 2001; Spillmann, 2001). The low affinities of some viral HS binding and its commonality between viruses with different tropisms have led to the idea that HS provides only non-specific virion adsorption, and is much less important for tropism than protein receptor engagements (Spillmann, 2001). However, some HS binding has high affinity (Rux *et al.*, 2002), and the high valencies of both cells and virions should make even low-affinity binding essentially irreversible. HS binding may therefore be an important determinant of how a virus colonizes its host.

Herpesviruses commonly express multiple HS-binding glycoproteins. For example, herpes simplex virus binds to HS via gB, gC and gD (Shukla & Spear, 2001), with some redundancy between gB and gC (Herold *et al.*, 1994; Laquerre *et al.*, 1998). Among the rhadinoviruses, murid herpesvirus-4 (MuHV-4) and the Kaposi's sarcoma-associated herpesvirus (KSHV) each has at least two HS-binding glycoproteins. MuHV-4 binds to HS via gp70 and gH/gL (Gillet *et al.*, 2007a, 2008a); KSHV does so via K8.1 (Birkmann *et al.*, 2001; Wang *et al.*, 2001), its gp70 homologue (Mark *et al.*, 2006), and possibly also gB (Akula *et al.*, 2001).

The MuHV-4 K8.1 homologue, gp150, has a more unusual HS interaction than simple binding. It binds to HS at best weakly (Gillet *et al.*, 2007a). Whilst wild-type (wt) MuHV-4 infection is highly HS-dependent, gp150 knockouts are hardly HS-dependent at all; for example they are 1000-fold less sensitive to inhibition by soluble heparin (de Lima *et al.*, 2004; Gillet *et al.*, 2007a). Gp150^−^ mutants are also relatively unaffected by antibody blocks to gH/gL and gp70 HS-binding (Gillet *et al.*, 2008a). Since gp150 mutants specifically gain binding to HS-deficient targets, gp150 must normally inhibit HS-independent cell binding. The weak HS interaction of gp150 presumably allows it to be displaced from its inhibitory site only when virions are already bound to cells, that is, after HS engagement by gH/gL or gp70 (Gillet *et al.*, 2008a). Thus, MuHV-4 cell binding, as studied *in vitro*, is entirely HS-dependent. Gp150^−^ virions are defective mainly in release from infected cells. This corresponds to infected cells having reduced HS expression (de Lima *et al.*, 2004): gp150^+^ virions readily escape, but gp150^−^ virions cannot.

Although HS binding is important for many herpesviruses *in vitro*, there has been little analysis of its importance *in vivo*. Removing HS from the host is technically difficult. The alternative strategy pursued here was to remove HS-binding proteins from the virus – the gH/gL and gp70 of MuHV-4. gH is essential for MuHV-4 infectivity, but gL is not; it is essential only for gH to adopt its HS-binding conformation (Gill *et al.*, 2006; Gillet *et al.*, 2007b). gL disruption therefore removes gH/gL-dependent HS-binding without rendering virions non-infectious. Gp70 functions in complement evasion (Kapadia *et al.*, 1999) as well as HS-binding, but is also non-essential (Adler *et al.*, 2000; Kapadia *et al.*, 2002). In contrast to gL^−^ and gp70^−^ single knockouts, MuHV-4 lacking both was severely impaired for cell binding and host entry. Both deficits were relieved by disrupting gp150. A major function of MuHV-4 HS binding is therefore to promote host entry.

## METHODS

### Mice.

Female C57BL/6 mice (Harlan UK Ltd) were infected intranasally with MuHV-4 when 6–8 weeks old (Home Office Project Licence 80/1992), using 300 plaque-forming units (p.f.u.) unless stated otherwise.

### Cells.

Hamster kidney (BHK-21) fibroblasts, NIH-3T3-CRE fibroblasts (Stevenson *et al.*, 2002) and NMuMG epithelial cells were grown in Dulbecco's modified Eagle medium supplemented with 2 mM glutamine, 100 U penicillin ml^−1^, 100 *μ*g streptomycin ml^−1^ and 10 % fetal calf serum (complete medium).

### Viruses.

All viruses were derived from an MuHV-4 bacterial artificial chromosome (BAC) (Adler *et al.*, 2000). Established mutations of gL (Gillet *et al.*, 2007b) and gp70 (Adler *et al.*, 2000; Gillet *et al.*, 2007a) were combined by introducing the gL^−^DEL^−^STOP mutation into the gp70^−^ BAC. This disrupted the gL coding sequence (genomic co-ordinates 65 437–65 024 of the murine herpesvirus 68 strain GenBank accession no. U97553) by deleting 65 362–65 437 and inserting in the same site of stop codons plus an *Eco*RI restriction site; the gp70 coding sequence (genomic co-ordinates 9873–11 039) was disrupted by deleting 9954–10 984 and inserting an *Eco*RI restriction site. We generated two independent double mutants. One was further modified by introducing the M7^−^STOP mutation (de Lima *et al.*, 2004) to make a gL^−^gp70^−^gp150^−^ triple mutant. Thus, stop codons plus an *Eco*RI site were inserted in an *Afe*I site (69 473) of the gp150 coding sequence (69 466–70 917). We also used an *Mlu*I–*Bgl*II genomic clone (Adler *et al.*, 2000) to revert the gp70 locus of the gL^−^gp70^−^ mutant BAC, thereby making it a single mutant (gL^−^REV). Infectious virus was reconstituted by transfecting BAC DNA into BHK-21 cells. For *in vivo* experiments, the *lox*P-flanked BAC/enhanced green fluorescent protein (eGFP) cassette was removed by passaging viruses through NIH-3T3-CRE cells. Virus stocks were grown in BHK-21 cells (May *et al.*, 2005a). Cell debris was pelleted by low speed centrifugation (400 ***g***, 10 min) and virions were recovered from the infected cell supernatants by ultracentrifugation (30 000 ***g***, 90 min). For some experiments, the virions were further purified by density-gradient centrifugation (May *et al.*, 2005a). This made no difference to the results.

### Viral infectivity assays.

Virus stocks were titrated by plaque assay (de Lima *et al.*, 2004). BHK-21 cell monolayers were incubated with virus dilutions (4 h, 37 °C), overlaid with 0.3 % carboxymethylcellulose, then 4 days later fixed in 4 % formaldehyde and stained with 0.1 % toluidine blue. We did not remove the input virus, so as to detect gL^−^gp70^−^ virions with maximum sensitivity by giving them maximum time to establish infection. When the input was removed after 2 h, gL^−^gp70^−^ plaque titres were markedly reduced. Infectious virus in lungs was measured by freezing and thawing the lungs and homogenizing them in complete medium. Tissue debris was pelleted (1000 ***g***, 1 min) and homogenate supernatants were titrated by plaque assay. Latent virus was measured by infectious centre assay (de Lima *et al.*, 2004): single-cell suspensions of spleens or mediastinal lymph nodes were co-cultured with BHK-21 cell monolayers, which were fixed and stained for plaque counting after 4 days. The plaque assay titres of freeze–thawed lymphoid tissue homogenates were always <1 % of infectious centre assay titres.

### Viral genome quantification.

MuHV-4 genomic co-ordinates 4166–4252 were amplified by real-time PCR of DNA extracted from mouse spleens (Rotor Gene 3000; Corbett Research). The PCR products were quantified by hybridization with a fluorescent probe matching genomic coordinates 4219–4189 and converted to genome copies by comparison with a standard curve of plasmid template amplified in parallel. Adenosine phosphoribosyltransferase mRNA was quantified as a cellular control (forward primer 5′-GGGGCAAAACCAAAAAAGGA-3′, reverse primer 5′-GCTGGAATTACCGCGGCT-3′, probe 5′-CGCAAATTACCCACTCCCGACCC-3′).

### Southern blotting.

Viral DNA was extracted by alkaline lysis (de Lima *et al.*, 2004), digested with restriction endonucleases, electrophoresed and transferred to nylon membranes (Roche Diagnostics). A [^32^P]dCTP-labelled probe (APBiotech) was generated by random primer extension (DECAprime II kit; Ambion) of cloned MuHV-4 genome segments (Efstathiou *et al.*, 1990). Membranes were hybridized with probe (65 °C, 18 h), washed to a stringency of 30 mM sodium chloride/3 mM sodium citrate/0.1 % SDS at 65 °C and exposed to X-ray film.

### ELISA.

Maxisorp ELISA plates (Nalgene Nunc) were coated (18 h, 4 °C) with 0.05 % Triton X-100-disrupted MuHV-4 virions. Plates were washed three times with PBS 0.1 % Tween-20, blocked with PBS 0.1 % Tween-20 1 % BSA, incubated with serum dilutions (1 h, room temperature), washed four times, incubated with alkaline phosphatase-conjugated goat anti-mouse IgG pAb, washed five times and developed with nitrophenylphosphate substrate (Sigma-Aldrich). The reaction was terminated with NaOH and the absorbance read at 405 nm (Bio-Rad Benchmark ELISA plate reader).

### Monoclonal antibodies (mAbs).

We used the following mAbs: 3F7, anti-gN IgG_2a_ (May *et al.*, 2005b); 7E5, anti-gH/gL IgG_2a_; T2C12, anti-gH/gL IgG_2a_; 8C1, anti-gH IgG_2b_ (Gill *et al.*, 2006); LT-6E8, anti-gp70 IgG_2b_ (Gillet & Stevenson, 2007a); LT-4D11, anti-gp70 IgG_2a_ (this study); T1A1, anti-gp150 IgG_2a_; T4G2, anti-gp150 IgG_2a_ (Gillet *et al.*, 2007d); BH-6H2, anti-gp150 IgG_1_ (this study); MG-4D11, anti-gB IgG_2a_; MG-12B8, ORF65 capsid component IgG_2a_ (Gillet *et al.*, 2006); 150-7D1, anti-ORF17 capsid component IgG_2a_ (Gillet & Stevenson, 2007b); 6D10, anti-gp48 IgG_2a_ (May *et al.*, 2005c).

### Immunoblotting.

Virions were denatured (95 °C, 5 min) in Laemmli's buffer, resolved by SDS-PAGE and proteins transferred to PVDF membranes (de Lima *et al.*, 2004). The membranes were probed with MuHV-4-specific mAbs plus horseradish peroxidase-conjugated rabbit anti-mouse IgG pAb (Dako Cytomation), followed by enhanced chemiluminescence substrate development (APBiotech).

### Flow cytometry.

Cells exposed to eGFP^+^ viruses were trypsinized, washed in PBS and analysed for green channel fluorescence. For surface staining, cells were incubated (1 h, 4 °C) with MuHV-4 glycoprotein-specific mAbs followed by Alexa 633-conjugated or Alexa 488-conjugated goat anti-mouse pAb (Invitrogen). For intracellular staining, cells were fixed in 1 % paraformaldehyde (30 min, room temperature) and permeabilized with 0.1 % saponin before staining. Cells were analysed on a FACSort using CellQuest (Becton Dickinson).

### Immunofluorescence.

Cells were exposed to MuHV-4 virions, then washed three times in PBS, fixed in 4 % paraformaldehyde, permeabilized with 0.1 % Triton X-100, and stained with MuHV-4-specific mAbs plus Alexa 488-conjugated goat anti-mouse IgG pAb (Invitrogen). Fluorescence was visualized with an Olympus IX70 microscope plus a Retiga 2000R camera line (QImaging).

## RESULTS

### Generation of MuHV-4 lacking both gL and gp70

**Fig. 1. f1:**
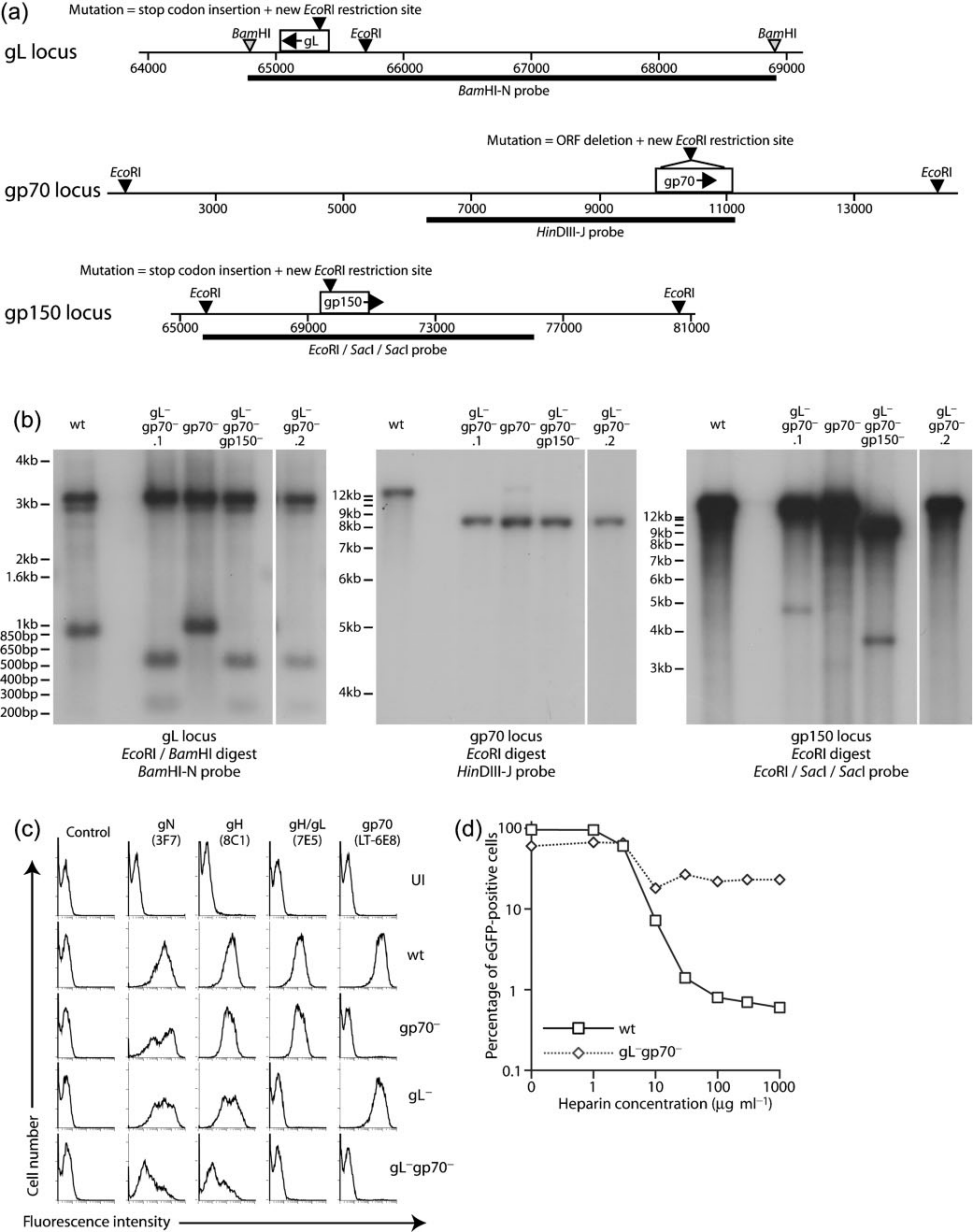
Generation of MuHV-4 mutants. In order to ablate expression, translational stop codons were inserted into the coding sequences of gL, gp70 and gp150 as indicated. Single gL and gp70 mutants have been described previously. (a) For this study, we combined gL and gp70 mutations, and then made a triple mutant by further disrupting gp150. (b) Viral DNA was analysed by Southern blotting, exploiting the novel *Eco*RI site introduced during mutagenesis. Wild-type (wt) and gp70^−^ viruses provide comparisons. gL^−^gp70^−^.1 and gL^−^gp70^−^.2 are independent mutants. For gL, there is a 3.1 kb invariant band and a 942 bp wt band that changes to 270 bp+595 bp. For gp70, a 12.7 kb wt band changes to 3.4 kb+8.4 kb. The 3.4 kb band was evident by ethidium bromide staining (data not shown), but not by hybridization because it overlaps the probe by only 115 bp. For gp150, a14.9 kb wt band changes to 11.2 kb+3.8 kb. (c) BHK-21 cells were left uninfected (UI) or infected (2 p.f.u. cell^−1^) with wt (18 h infection), single mutant (gL^−^, gp70^−^, 18 h infections) or double mutant viruses (gL^−^gp70^−^, 66 h infection), then analysed by flow cytometry for surface expression of virion glycoproteins. (d) BHK-21 cells were exposed to BAC^+^ wt (1 p.f.u. cell^−1^) or gL^−^gp70^−^ (5 p.f.u. cell^−1^) viruses plus soluble heparin. Both viruses expressed eGFP from an HCMV IE1 promoter, and infection was quantified by flow cytometry after 24 h. Different virus doses were used to ensure comparable eGFP expression. The difference in susceptibility to heparin was much greater than the difference in virus input.

**Fig. 2. f2:**
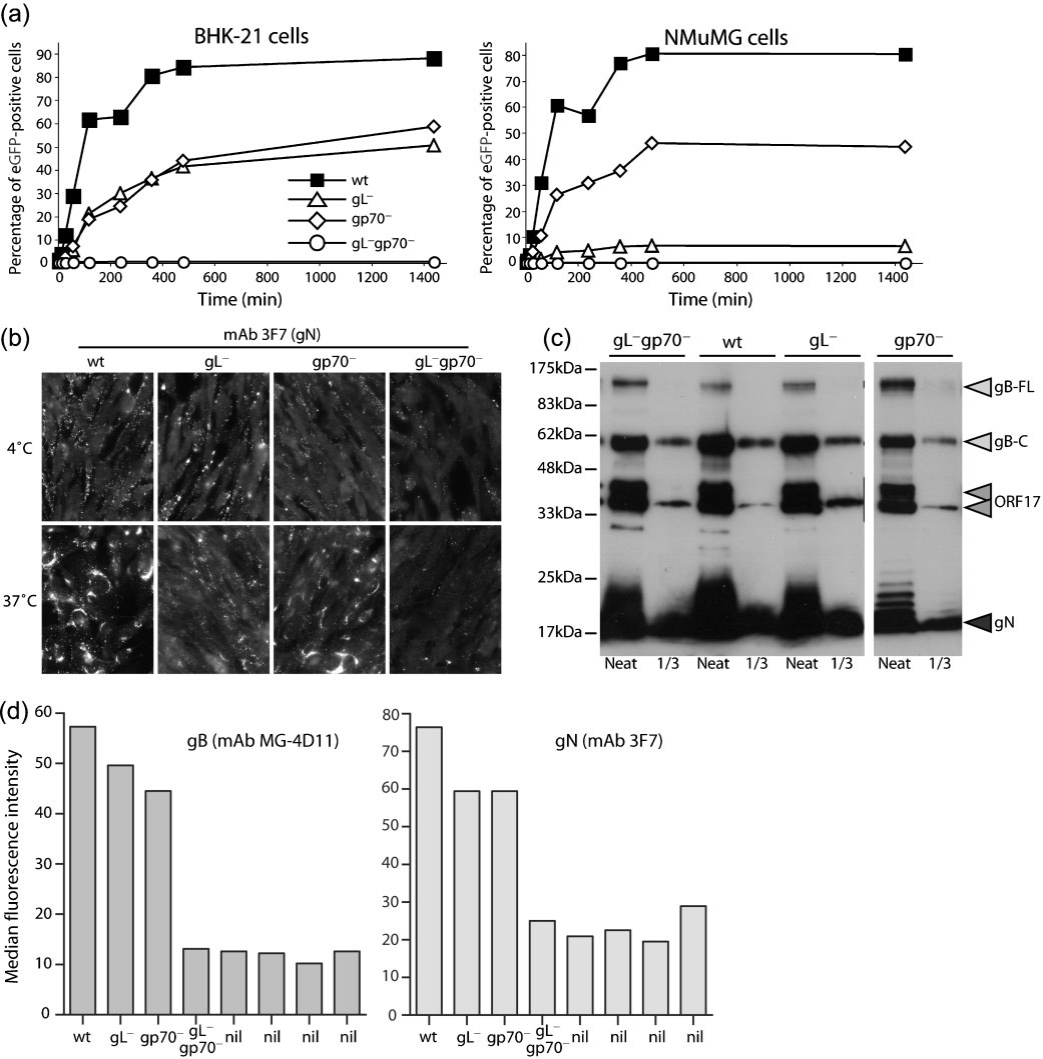
gL^−^gp70^−^ MuHV-4 shows a severe deficit in cell binding. (a) BHK-21 or NMuMG cells were exposed to viruses (1 p.f.u. cell^−1^, 37 °C) for different times, then washed three times with PBS to remove unbound virions. After 24 h, all cells were analysed by flow cytometry for viral eGFP expression. Each point shows 2×10^4^ cells. (b) NMuMG cells were exposed to wt or mutant viruses (2 h, 4 °C, 5 p.f.u. cell^−1^), then washed three times with PBS and either fixed immediately (4 °C) or first incubated in complete medium (2 h, 37 °C). All the cells were then stained for gN, an abundant component of the virion envelope. New gN expression is not evident until at least 6 h p.i., so this assay detects only input virus. (c) Virus stocks were compared by immunoblot for gB (mAb MG-4D11), ORF17 (mAb 150-7D1) and gN (mAb 3F7). gB-FL, full-length gB; gB-C, C-terminal cleavage product. ORF17 is auto-cleaved and so appears as a doublet. (d) NMuMG cells were exposed to wt or mutant viruses (2 h, 37 °C, 3 p.f.u. cell^−1^), then washed three times with PBS and analysed for virion binding by fixation, permeabilization and staining for gB or gN. nil, No virus. Each bar shows 2×10^4^ cells. Fixation/permeabilization was used to make virion detection independent of endocytosis.

A MuHV-4 BAC mutated for gp70 was modified further by disrupting the gL and gp150 coding sequences (Fig. 1a[Fig f1]). Southern blot analyses (Fig. 1b[Fig f1]) demonstrated that the viruses generated contained the desired genomic changes. Flow cytometry of infected cells (Fig. 1c[Fig f1]) confirmed an absence of gp70 or gL expression in the cells infected with the gL^−^gp70^−^ double mutant. Although gL^−^gp70^−^ mutants were viable, they replicated poorly in BHK-21 cells: virus stocks typically required 2–3 weeks to grow rather than 4–5 days. The weak surface gN and gH staining of cells infected with MuHV-4 gL^−^gp70^−^ (Fig. 1c[Fig f1]) was consistent with poor infectivity. It also suggested poor cell binding, as virions bound on the plasma membrane probably account for much of the virion antigen detected on infected cells (de Lima *et al.*, 2004). There was no sign of gL^−^gp70^−^ mutants containing less gN than the wt (see Figs 2c[Fig f2] and 6a[Fig f6]), so there did not appear to be a general problem with virion assembly or export.

Wild-type MuHV-4 is readily inhibited by soluble heparin, presumably because this competes with cellular HS for binding to gH/gL and gp70 (Gillet *et al.*, 2008a). The gL^−^gp70^−^ mutant, lacking HS binding, was accordingly less sensitive to such inhibition (Fig. 1d[Fig f1]). What inhibition there was presumably reflected the role of HS in displacing virion gp150 (de Lima *et al.*, 2004; Gillet *et al.*, 2007a, 2008a). This function was evidently less sensitive to inhibition by soluble heparin than were gH/gL-dependent or gp70-dependent HS binding, consistent with the inhibition of wt infection by heparin being mainly an inhibition of cell binding (Gillet *et al.*, 2008a).

### Poor cell binding by gL^−^gp70^−^ MuHV-4

Although gL^−^gp70^−^ MuHV-4 could form plaques, infection was clearly delayed relative to wt. Assays of viral eGFP expression at 24 h post-infection (p.i.) showed a marked gL^−^gp70^−^ deficit compared with wt or single-gene mutants (Fig. 2a[Fig f2]). eGFP expression was used to measure infection because it is independent of MuHV-4 lytic gene expression (Smith *et al.*, 2007), which can be variable (May *et al.*, 2004), and because it operates over 4–6 h rather than 4 days, making it intrinsically better at detecting infection delays. There was no evidence of eGFP expression under-recording gL^−^gp70^−^ infection, as infected cultures always contained more eGFP^+^ cells than glycoprotein^+^ cells. NMuMG cells were infected poorly by both gL^−^ and gL^−^gp70^−^ mutants, indicating that for this cell type the deficit was mainly gL-dependent. But BHK-21 cells showed only twofold less infection for the gL^−^ and gp70^−^ single mutants compared with wt, and more than 100-fold less for gL^−^gp70^−^ mutant.

MuHV-4 gL knockouts have a cell-binding deficit mainly for BHK-21 cells (Gillet *et al.*, 2007b); their poor infection of NMuMG cells reflects instead a post-binding entry deficit (Gillet *et al.*, 2008b). We therefore used NMuMG cells to define the effect on cell binding of a combined gL/gp70 disruption (Fig. 2b[Fig f2]). The cells were incubated with virions for 2 h at 4 °C, washed three times with PBS, then either fixed immediately or first incubated for a further 2 h at 37 °C to allow endocytosis and virion uncoating. The fixed, permeabilized cells were then stained for gN to quantify total virion binding (bound virions still at the cell surface plus bound virions that were subsequently endocytosed). This assay measures only virion binding/uptake, as new glycoprotein expression is undetectable at 4 h p.i. (Gill *et al.*, 2006). The gN staining of the gL^−^gp70^−^ mutant was much reduced, while that of the gL^−^ and gp70^−^ single mutants was close to wt.

Binding was tested further using virus stocks normalized by immunoblot for gB, gN and the ORF17 capsid component rather than by plaque titre. A sample immunoblot showing approximately equal inputs is shown in Fig. 2(c) (gL^−^gp70^−^ stocks had at most a threefold increase in virion protein content p.f.u.^−1^, confirming that the plaque assay provided a reasonably sensitive measure of gL^−^gp70^−^ infectivity). NMuMG cells were incubated with virions for 2 h at 37 °C, then washed three times in PBS, fixed, permeabilized and stained for virion uptake with mAbs MG-4D11 (gB) and 3F7 (gN) (Fig. 2d[Fig f2]). Again, cell binding by the gL^−^gp70^−^ double mutant was hardly detectable, whereas that of the single mutants was relatively unimpaired.

### Infection of mice with MuHV-4 lacking both gL and gp70

**Fig. 3. f3:**
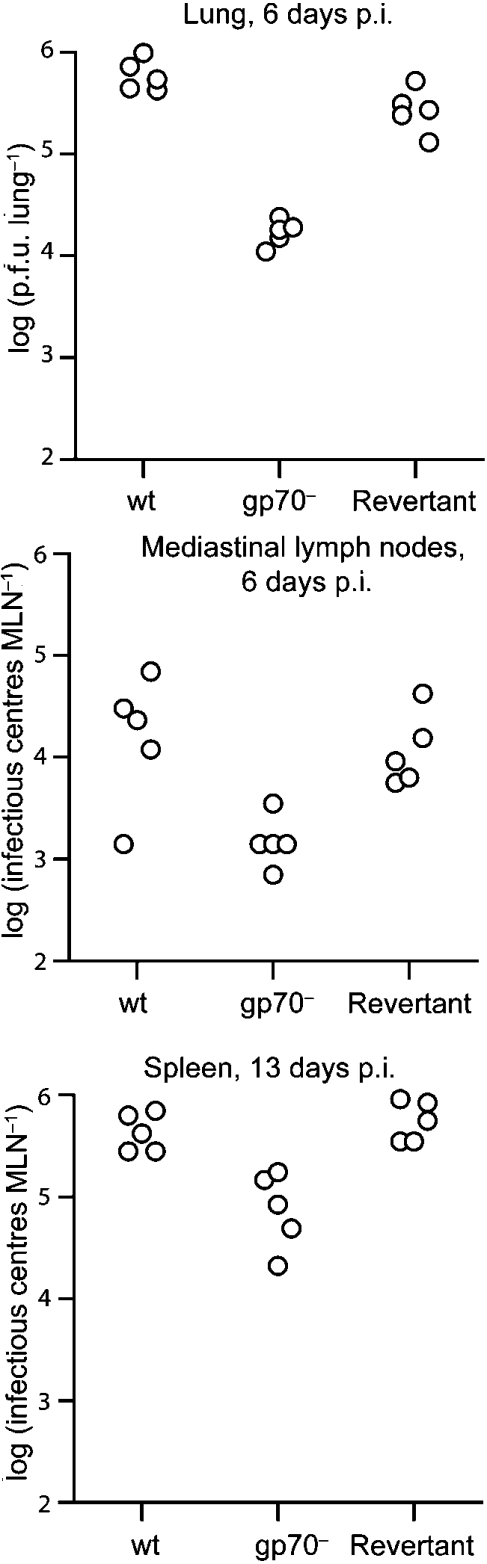
*In vivo* replication of gp70^−^ MuHV-4 after intranasal inoculation. Mice were infected intranasally (300 p.f.u.) with wt, gp70^−^ or revertant (gp70^+^) viruses, then analysed for infectious virus in lungs by plaque assay and for latent virus in lymphoid tissue by infectious centre assay. Each point shows the titre of one mouse. gp70^−^ virus titres in lungs and spleens were significantly reduced compared with wt (*P*<0.001 by Student's *t*-test). Although the gp70^−^ mediastinal lymph node titres were also lower, the difference did not reach statistical significance (*P*=0.06).

Gp70 disruption has been linked to a lytic replication deficit after intracerebral or intraperitoneal MuHV-4 inoculation (Kapadia *et al.*, 2002). However, intranasal infection has not been studied, and the gp70 mutant used here has been characterized only *in vitro* (Adler *et al.*, 2000). Before analysing the gL^−^gp70^−^ mutant, therefore, we tested the gp70^−^ single mutant for host colonization after intranasal inoculation (Fig. 3). Lytic replication was measured by plaque assay of lungs at 6 days p.i.; latency establishment was measured by infectious centre assay of mediastinal lymph nodes at 6 days p.i. (seeding) and of spleens at 13 days p.i. (peak titres). The gp70^−^ mutant showed reduced lytic replication in the lung, reduced seeding to lymph nodes, and reduced peak latency titres in the spleen. These results did not distinguish the gp70 functions of complement evasion and HS binding, but established that gp70 disruption alone causes a moderate defect in host colonization by the intranasal route. We have shown before that intranasally delivered gL^−^ MuHV-4 spreads and establishes latency much like wt (Gillet *et al.*, 2007b).

**Fig. 4. f4:**
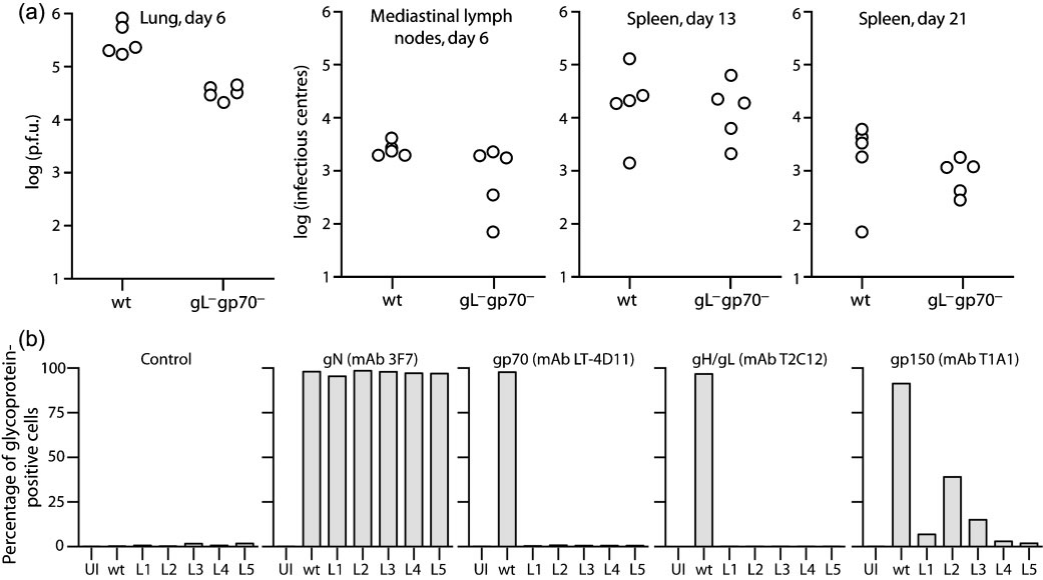
*In vivo* infection with gL^−^gp70^−^ MuHV-4. (a) Mice were infected intranasally (300 p.f.u.) with wt or gL^−^gp70^−^ MuHV-4, then analysed for infectious virus in lungs by plaque assay and for latent virus in lymphoid tissue by infectious centre assay. Each point shows the titre of one mouse. gL^−^gp70^−^ titres were significantly lower than wt in lungs at day 6 (*P*<0.001 by Student's *t*-test) but not in lymphoid tissue. (b) gL^−^gp70^−^ viruses from the individual mouse lungs in (a) (L1-L5) were propagated in BHK-21 cells for 7 days then analysed for glycoprotein expression by flow cytometry. Cells were scored as stained or not, based on a gate excluding >99 % of uninfected cells. Uninfected (UI) and wt virus-infected (wt) BHK-21 cells provided controls. Each bar shows 2×10^4^ cells. For equivalent gN expression, gp150 expression by the gL^−^gp70^−^ knockouts was significantly reduced compared with wt (*P*<10^−5^ by *χ*^2^ test).

We then infected mice with gL^−^gp70^−^ MuHV-4 and compared its replication with wt (Fig. 4a[Fig f4]). Surprisingly, there was only a deficit in lytic replication, and this was no greater than with the gp70^−^ single mutant. The latent viral load in mediastinal lymph nodes and spleens was normal. gL^−^gp70^−^ viruses recovered from infected lungs appeared to grow better *in vitro* than the original mutant (data not shown). We therefore propagated these viruses further and analysed them for glycoprotein expression (Fig. 4b[Fig f4]). BHK-21 cells were infected with either the gL^−^gp70^−^ viruses recovered from infected lungs (L1-L5) or with wt MuHV-4 as a control. As cells infected with wt express all virion glycoproteins more strongly than those infected with gL^−^gp70^−^ mutants (Fig. 1c[Fig f1]), we measured each glycoprotein by the number of positively staining cells compared with the number positive for gN, an essential virion component. There was no sign of the L1-L5 mutants regaining gL or gp70 expression, but each showed much reduced gp150 expression.

DNA sequence analysis established that the gp150-coding sequence of the recovered viruses (genomic co-ordinates 69 466–70 917) was disrupted by a deletion of co-ordinates 70 020–70 847 and the insertion of co-ordinates 91 761–91 866, duplicated from ORF64. Thus, gp150 translation was terminated upstream of its membrane anchor. The same mutation was present in all mice, and was found to be a minor contaminant of the BAC^−^ inoculating virus, but not the BAC^+^ parent analysed in Figs 1 and 2. Thus, it arose during gL^−^gp70^−^ virus passage in NIH-3T3-CRE cells and was then strongly selected during *in vivo* infection and *ex vivo* virus recovery. It was not visible (a predicted 722 bp deletion of the 14.9 kb wt *Eco*RI band) in the gL^−^gp70^−^.1 Southern blot of Fig. 1(b). The outgrowth of the gL^−^gp70^−^gp150^−^ mutant supported the idea that gL^−^gp70^−^ mutants were attenuated due to a loss of HS binding, as removing gp150 offsets such a loss (Gillet *et al.*, 2008a).

### Comparison of gL^−^gp70^−^ and gL^−^gp70^−^gp150^−^ MuHV-4 mutants

**Fig. 5. f5:**
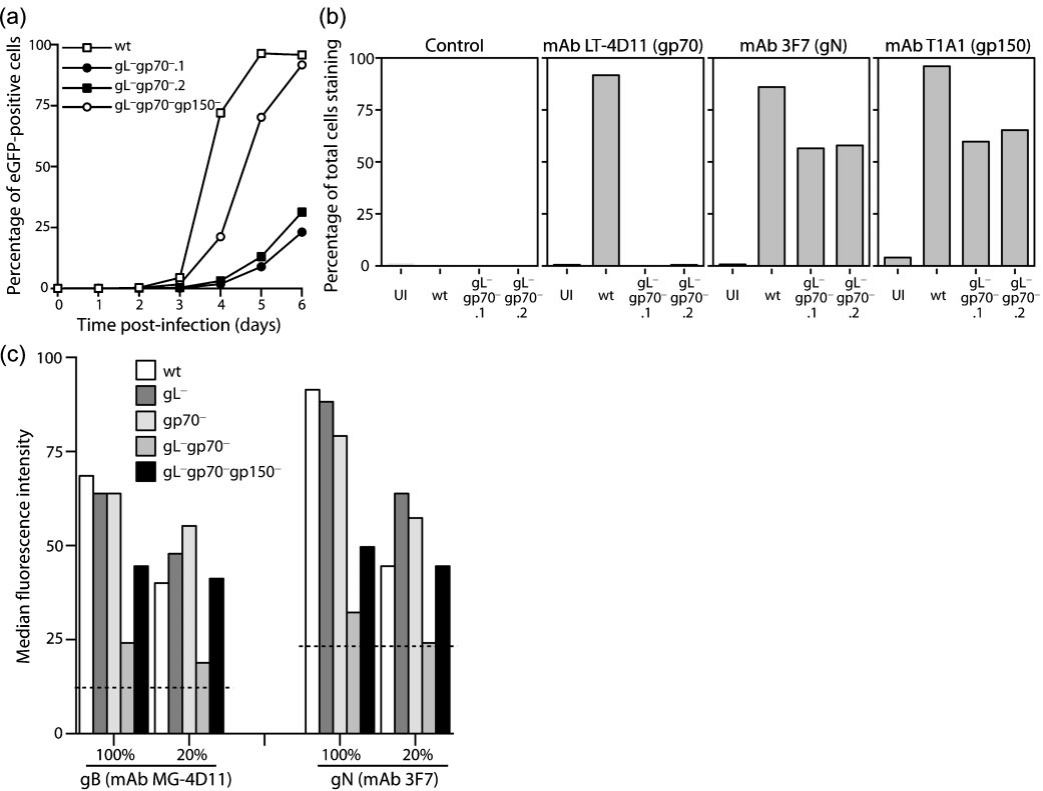
*In vitro* growth of gL^−^gp70^−^ MuHV-4. (a) BHK-21 cells were infected (0.01 p.f.u. cell^−1^) with eGFP-expressing forms of each virus, then monitored by flow cytometry of eGFP expression. Each point shows 2×10^4^ cells. (b) gL^−^gp70^−^ infected cells from (a) were then analysed for viral glycoprotein expression. Cells uninfected (UI) or infected overnight with wt MuHV-4 (1 p.f.u. cell^−1^) provided controls. For both the gL^−^gp70^−^ mutants, gp150 expression was equivalent to gN expression. (c) BHK-21 cells were exposed to virus stocks normalized by immunoblot (2 h, 37 °C). The equivalent infectivity for wt was 3 p.f.u. (100 %) or 0.6 p.f.u. (20 %). The cells were washed three times with PBS to remove unbound virions, fixed, permeabilized and stained for gN or gB. The horizontal dashed lines show the fluorescence of uninfected cells analysed in parallel. Each bar shows the result for 2×10^4^ cells. Uptake of the gL^−^gp70^−^gp150^−^ mutant was significantly higher than the gl^−^gp70^−^ mutant even with 1/5 the input (*P*<10^−5^ by Student's *t*-test).

In order to identify more definitively the gL^−^gp70^−^ *in vivo* phenotype and the impact on it of gp150 disruption, we compared a second gL^−^gp70^−^ mutant with no evidence of gp150 disruption (gL^−^gp70^−^.2) with both wt MuHV-4 and a deliberately designed gL^−^gp70^−^gp150^−^ triple mutant (Fig. 5). The triple mutant grew much better *in vitro* than either the gL^−^gp70^−^.2 mutant or the BAC^+^ form of gL^−^gp70^−^.1, which retained gp150 (Fig. 5a[Fig f5]). Flow cytometry of the infected cultures after 1 week (Fig. 5b[Fig f5]) established that both gL^−^gp70^−^ mutants remained gp150^+^: the number of gp150^+^ cells was equivalent to the number of gN^+^ cells. Cell-binding experiments (Fig. 5c[Fig f5]) similar to those in Fig. 2 established that gL^−^gp70^−^gp150^−^ MuHV-4 was much less impaired for cell binding than its gL^−^gp70^−^ parent. Removing gp150 therefore improved markedly both the cell binding and the *in vitro* propagation of gL^−^gp70^−^ MuHV-4.

### *In vivo* comparison of gL^−^gp70^−^ and gL^−^gp70^−^gp150^−^ MuHV-4

**Fig. 6. f6:**
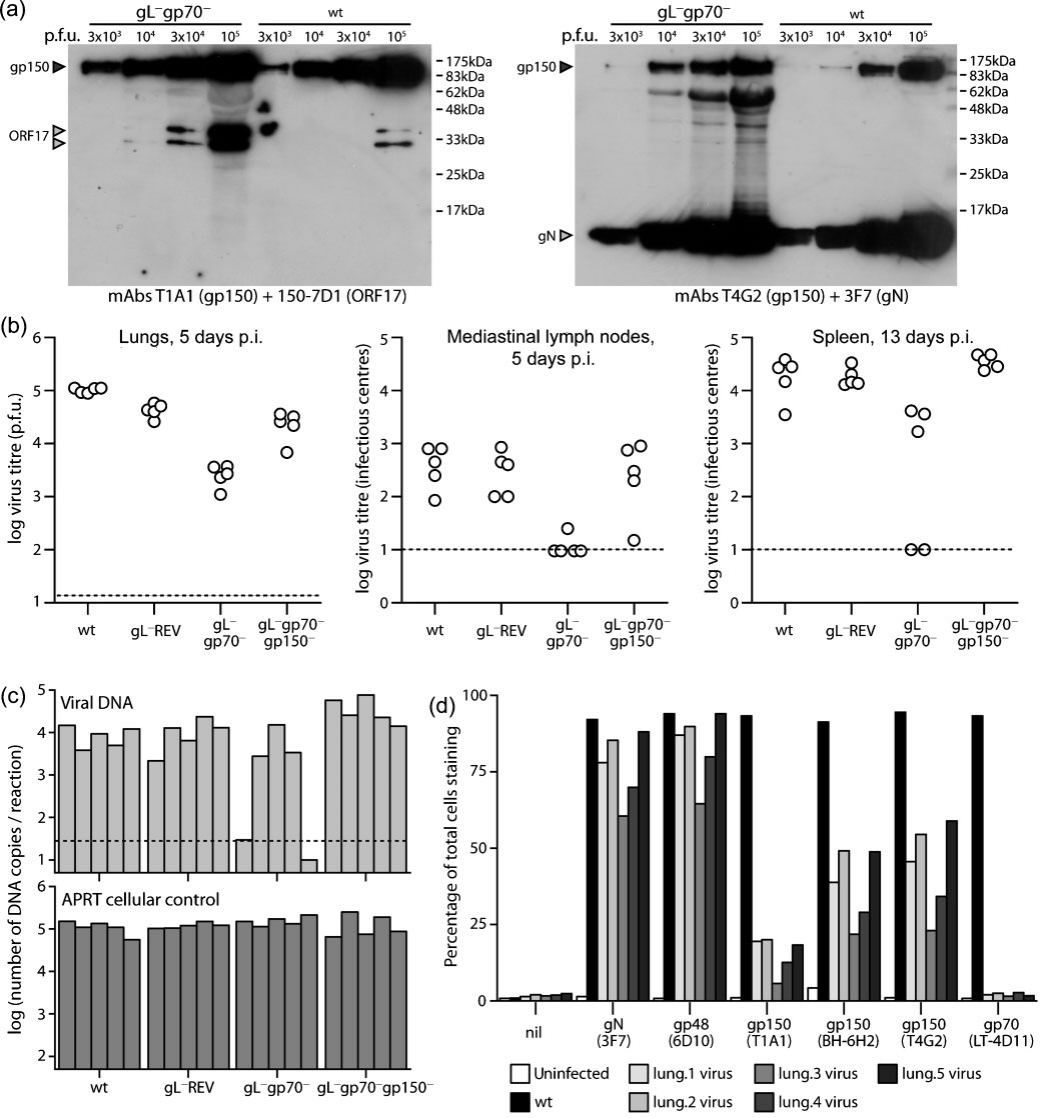
*In vivo* infection with gL^−^gp70^−^gp150^+^ MuHV-4. (a) The gL^−^gp70^−^.2 mutant was analysed for gp150 content by immunoblot with mAbs that recognize gp150 residues 152–269 (T1A1) or 108–151 (T4G2) (Gillet *et al.*, 2007d). gN and the ORF17 capsid component provided loading controls. The 62 kDa band in the T4G2+3F7 immunoblot of the gL^−^gp70^−^ stock is non-specific staining of bovine albumin. Because gL^−^gp70^−^ stocks had lower titres than wt, more albumin was present at comparable infectivity. (b) Mice were infected intranasally (300 p.f.u.) with wt or gL^−^gp70^−^ viruses analysed in (a), then titrated for infectious virus in lungs by plaque assay and for latent virus in lymphoid tissue by infectious centre assay. Each point shows the titre of one mouse. The horizontal dashed lines show lower limits of detection. gL^−^gp70^−^, but not gL^−^gp70^−^gp150^−^ virus titres were significantly lower than wt in all sites (*P*<0.01 by Student's *t*-test). (c) DNA was extracted from the spleens in (b) and viral genome copy numbers determined by real-time PCR. Each bar shows the average of triplicate reactions. The horizontal dashed line shows the average of three uninfected controls. (d) gL^−^gp70^−^ viruses were recovered from infected lungs by propagation in BHK-21 cells for 14 days, then analysed for virion glycoprotein expression by flow cytometry. BHK-21 cells either uninfected or infected overnight with wt MuHV-4 (1 p.f.u. cell^−1^, 18 h), provided staining controls. nil, Secondary antibody only. Each bar shows 2×10^4^ cells. mAbs T4G2, T1A1 and BH-6H2 recognize different gp150 epitopes.

We then analysed a BAC^−^ form of the gL^−^gp70^−^.2 mutant *in vivo* (Fig. 6). The gp150 coding sequence of this mutant was confirmed as wt by PCR and sequencing of viral DNA. Immunoblots of virion lysates (Fig. 6a[Fig f6]) showed that the gL^−^gp70^−^ mutant stock had approximately three times the protein content of wt for an equivalent number of p.f.u., but for an equivalent amount of ORF17 (capsid) or gN, the gp150 content of wt and gL^−^gp70^−^ mutant viruses was similar. Therefore, there was no sign of the gL^−^gp70^−^.2 mutant being rescued by spontaneous gp150 loss.

As with the gL^−^gp70^−^.1 mutant, the gL^−^gp70^−^.2 mutant showed an acute lytic replication deficit in infected lungs (Fig. 6b[Fig f6]). It also showed a latency deficit in lymphoid tissue, like the original gp70 single knockout (Fig. 3), whereas gL**^−^**gp70**^+^** and gL**^−^**gp70**^−^**gp150**^−^** viruses did not (thus, the lack of a latency deficit for the gL^−^gp70^−^.1 mutant could be attributed to the spontaneous loss of gp150). Interestingly, two out of five gL^−^gp70^−^.2-exposed mice showed no detectable infection by infectious centre assay at 13 days. Similar results were obtained by real-time PCR of viral genomes (Fig. 6c[Fig f6]), indicating that the lack of detectable infection was a failure of host colonization rather than a failure of *ex vivo* reactivation.

### Recovered gL^−^gp70^−^ viruses again show evidence of gp150 loss

We again analysed the glycoprotein expression of viruses recovered from infected lungs (Fig. 6d[Fig f6]). Although gp150 expression by the input gL**^−^**gp70**^−^** virus had been normal (Figs 5b[Fig f5], 6a[Fig f6]), that of the recovered viruses was again reduced. The degree of reduction varied between gp150-specific mAbs, but each showed fewer positive cells than gN-specific and gp48-specific mAb controls, whereas for wt the numbers were similar. This time, the gp150 coding sequence and its upstream 50 bp were entirely normal by PCR and DNA sequencing. There may have been changes in other virion components to reduce gp150 incorporation. Whatever the mechanism, it was clear that a lack of gL and gp70 strongly selected for gp150 loss.

### gL^−^gp70^−^ MuHV-4 shows reduced host entry

**Fig. 7. f7:**
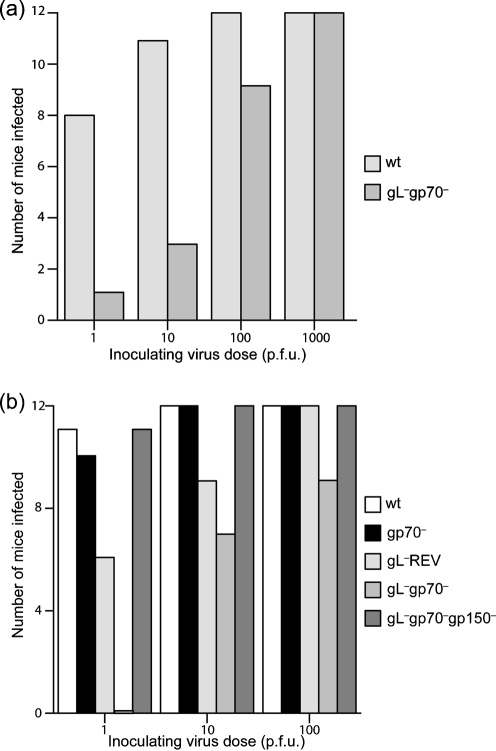
Low dose *in vivo* infection with gL^−^ and gp70^−^ viruses. (a) The gL^−^gp70^−^.2 mutant was compared with wt MuHV-4 by intranasal inoculation of mice with 1–1000 p.f.u. Twelve mice per group for each dose were scored as infected or not at 16 days p.i. by infectious centre assay of spleens and by ELISA for virus-specific serum IgG, with concordant results. The gL^−^gp70^−^ infection rate was significantly lower than wt at 1–10 p.f.u. (*P*<0.005 by Fisher's exact test), but not at 100 p.f.u. (*P*=0.1). (b) Virus knockouts were compared with wt for infectivity (12 mice per group) after intranasal inoculation. The gL^−^REV and gL^−^gp70^−^gp150^−^ mutants were derived from the gL^−^gp70^−^ mutant. Their infectivities were equivalent to wt. The gL^−^ and gL^−^gp70^−^ infection rates were both significantly less than wt at 1 p.f.u. (*P*<0.05 by Fisher's exact test). Only the gL^−^gp70^−^ infection rate was significantly lower at 10 p.f.u. (*P*<0.02). At 1 p.f.u. the gL^−^gp70^−^ infection rate was significantly lower than that of the gL^−^ (*P*<0.01).

Viruses are selected by their capacity to transmit, so a key parameter of evolutionary fitness is efficient entry into naive hosts. Wild-type MuHV-4 productively infects mice after intranasal inoculation of 1 p.f.u. (Gillet *et al.*, 2007c). The failure of some mice to become detectably infected after exposure to 300 p.f.u. of the gL^−^gp70^−^ mutant therefore suggested that HS binding might have an important role in host entry. To establish this more definitively, we tested whether low dose gL^−^gp70^−^ inoculations established infection (Fig. 7a[Fig f7]). Approximately 100 times more gL^−^gp70^−^ virus than wt was required.

We then compared the gL^−^gp70^−^ mutant with gp70^−^ and gL^−^ single mutants and with the gL^−^gp70^−^gp150^−^ triple mutant (Fig. 7b[Fig f7]). Again, 100-fold more gL^−^gp70^−^ virus than wt was required to establish infection. The gL^−^REV mutant also showed significantly less infection than wt at 1 p.f.u. per mouse, indicating a gL-dependent deficit, but at 1 p.f.u. gL^−^gp70^−^ deficit was significantly greater than that of gL^−^ REV, and at 10 p.f.u. only the gL^−^gp70^−^ infection was significantly less than wt. The gp70^−^ mutant showed no deficit. gp150 disruption completely restored efficient host entry to the gL^−^gp70^−^ mutant. The requirements for host entry therefore mirrored those for *in vitro* cell binding: one or more of the virion HS-binding proteins was required, unless gp150 was also missing.

## DISCUSSION

MuHV-4 infection of epithelial cells is highly HS-dependent (Gillet *et al.*, 2007a). mAb blocking and viral mutagenesis (Gillet *et al.*, 2008a) show two levels of dependence: first, virions must attach to HS via either gH/gL or gp70; second, HS interactions remove the inhibition that gp150 exerts over HS-independent cell binding. The gH/gL and gp70 interactions occur first, as a combined mAb block of gH/gL and gp70 only modestly affects gp150^−^ mutants, but severely attenuates wt MuHV-4 (Gillet *et al.*, 2008a). These results predicted that a cell-binding deficit for gL^−^gp70^−^ mutants would be offset by disrupting gp150, and *in vitro* infection studies supported this hypothesis. Infecting mice with gL^−^gp70^−^ and gL^−^gp70^−^gp150^−^ mutants then allowed us to link HS binding to host colonization. A lack of HS binding had a limited impact on viral dissemination, which depends mainly on latency-associated lymphoproliferation (Coleman *et al.*, 2003; Stevenson *et al.*, 1999), but severely reduced host entry.

Where might HS binding occur in host entry? Incoming virions arrive at mucosal epithelia, which carry HS on syndecans and glypicans (Bernfield *et al.*, 1999). The HS on syndecans is more distal to the membrane and therefore more likely to participate in virion capture. In support of this, overexpressing syndecan-1 on B cells increases their susceptibility to MuHV-4 infection (Bennett *et al.*, 2005). However, syndecan expression is confined to the basolateral side of confluent epithelial cells *in vitro* (Rapraeger *et al.*, 1986) and is lost entirely from differentiated epithelial cells *in vivo* (Hayashi *et al.*, 1987). Thus, while virions exiting a host could readily bind to basolateral epithelial HS, it is far from clear that the same interaction is available to incoming virions on the apical side. Human cytomegalovirus shows an HS-dependent restriction of apical epithelial infection *in vitro* (Esclatine *et al.*, 2001). MuHV-4 also infects confluent epithelial monolayers poorly *in vitro* (data not shown). Thus, incoming virions may require epithelial transcytosis, for example via M cells (Wolf *et al.*, 1981; Morin *et al.*, 1994), to interact with HS.

Transcytosis could also explain how intranasally delivered, replication-deficient MuHV-4 mutants can infect B cells (Kayhan *et al.*, 2007; Moser *et al.*, 2006). The MuHV-4 gp150 normally inhibits B cell infection (de Lima *et al.*, 2004). Its positional homologue in Epstein–Barr virus, gp350, analogously inhibits epithelial infection; this is overcome by gp350 binding to B cells (Shannon-Lowe *et al.*, 2006). The equivalent ‘transfer infection’ for MuHV-4 would be gp150 displacement by virion binding to epithelial HS. Thus, incoming MuHV-4 virions could be transported to the basolateral epithelium by M cells, engage HS, then either infect the epithelium itself from underneath or be transferred to submucosal B cells. Without gH/gL or gp70, virions would fail to engage epithelial HS, so the inhibitory effect of gp150 could not be undone, making host entry inefficient. With gp150 removed (the gL^−^gp70^−^gp150^−^ mutant), HS engagement might not be required. The disadvantage of gp150 deficiency, apart from reduced antibody evasion (Gillet *et al.*, 2007d), would be in host exit: virions lacking gp150 would be recaptured by HS-independent binding to the apical epithelium.

The latency deficit of gp70^−^, but not gL^−^, MuHV-4 after intranasal infection suggested that the redundancy of gH/gL and gp70 for epithelial HS-binding might be less elsewhere. For example, gp70 could have a particular role in promoting B-cell infection. Syndecan-1 is expressed only on pro-B cells and plasma cells, neither of which is a major target for MuHV-4, but syndecan-4 is widely expressed (Yamashita *et al.*, 1999) and gp70-Fc shows heparin-dependent binding to B cells (data not shown). A role for gp70 in B cell infection would be consistent with the lack of a latency deficit for gL^−^gp70^−^gp150^−^ MuHV-4: gp150 removal is unlikely to compensate for a lack of complement evasion, but could compensate for a lack of HS binding (Gillet & Stevenson, 2007b). Thus, it appears with MuHV-4 that HS interactions are not just a prelude to specific receptor binding, but rather a fundamental feature of virion tropism and host entry.
